# Monitoring the gasification area and its behavior in underground coal gasification by acoustic emission technique instead of temperature measurement

**DOI:** 10.1038/s41598-023-36937-0

**Published:** 2023-06-16

**Authors:** Akihiro Hamanaka, Yuma Ishii, Ken-ichi Itakura, Takashi Sasaoka, Hideki Shimada, Nuhindro Priagung Widodo, Budi Sulistianto, Jun-ichi Kodama, Gota Deguchi

**Affiliations:** 1 Department of Earth Resources Engineering, Kyushu University, Fukuoka, 819-0395 Japan; 2 Endowed Research Lab. of Un-mined Mineral Resources and Energy Eng., Muroran Institute of Technology, Muroran, 050-8585 Japan; 3 Department of Mining Engineering, Faculty of Mining and Petroleum Engineering, Institut Teknologi Bandung, Bandung, 40132 Indonesia; 4 Division of Sustainable Resources Engineering, Hokkaido University, Sapporo, 060-8628 Japan; 5 Underground Resources Innovation Network, Sapporo, 007-0847 Japan

**Keywords:** Coal, Acoustics

## Abstract

Underground Coal Gasification (UCG) requires monitoring of the gasification area because the gasification process is invisible and the reaction temperature exceeds 1000 °C. Many fracturing events that occurred due to coal heating can be captured with Acoustic Emission (AE) monitoring technique during UCG. However, the temperature conditions to generate fracturing events during UCG have not yet been clarified. Therefore, the coal heating experiment and small-scale UCG experiment are conducted by measuring the temperature and AE activities in this research to examine the applicability of the AE technique instead of temperature measurement as a monitoring method during UCG. As a result, many fracturing events are generated when the temperature of coal is changed drastically, especially during coal gasification. Besides, AE events increase in the sensor near the heat source and AE sources are expanded widely with the expansion of the high-temperature region. AE monitoring is an effective technique for the estimation of the gasification area during UCG instead of temperature monitoring.

## Introduction

The demand for energy increases day by day with economic and population growth worldwide. Coal is still an important energy resource as the primary energy due to the large reserves and less regional ubiquity. However, many coal resources are being left without excavation due to technological and economic reasons in conventional mining systems. Underground Coal Gasification (UCG) is a technique to recover coal energy from underground by in-situ gasification. UCG contributes to improving the energy recovery ratio from coal because it enables the recovery of energy from untapped coal. Additionally, the injection agents and temperature of the coal seam affect the product gas composition, e.g., the steam injection enhances hydrogen production^1–3^. Therefore, UCG is a promising option to improve the recovery of coal energy as a clean coal technology. In UCG, the product gas can be enhanced by expanding the gasification area with high temperatures. On the other hand, the excessive expansion of the gasification area causes several environmental problems such as gas leakage, deformation of the surrounding ground, and underground water pollution^4–6^. Therefore, UCG requires a monitoring system to control the gasification area.

The gasification area of UCG can be assumed with the temperature of the coal^7,8^. However, it is practically difficult to measure the temperatures of coal seams in the actual UCG site. Instead of temperature measurement, some techniques have been introduced to monitor the gasification area of UCG. Application of geophysical monitoring techniques has been discussed such as electrical resistance tomography^9^, electrical resistivity method^10^, microgravity survey^11^, and ground penetrating radar^12,13^. The estimation of the cavity growth and the velocity of gasification flame by means of a mathematical model has been also described^14–17^. This research focuses on the application of acoustic emission (AE) to monitor the gasification area. AE monitoring can be an alternative technique to temperature measurement because the micro-seismic will be occurred due to the expansion or contraction of coal under heating. AE generates simultaneously when the material generates fractures, meaning that real-time monitoring can be achieved by AE monitoring. Besides, it is possible to identify the location of fracturing activity by calculating the difference in seismic arriving time and sensor coordination with source location analysis. Many scholars have reported the generation of fracture with coal heating due to the different thermal expansion coefficients of minerals^18,19^, changing the pore structure^20–22^, and thermal contraction^23–25^. Ding et al.^26^ measured the AE count and the evolution of cracks with coal heating. AE signal is active especially when the crack width expands under 300–500 °C. It has been also reported that AE signals increase with heating under the loading condition compared to without heating^27^.

Our previous works have also indicated the possibility to utilize the AE technique as a monitoring method of the gasification area during the UCG process^28–31^. However, it is still uncertain the temperature conditions that these AE activities occur during the UCG process. This study discusses the applicability of the AE technique instead of temperature measurement as monitoring of gasification area by means of coal heating experiment and small-scale UCG experiment.

## Materials and methods

### Coal heating experiment

**Figure 1:**
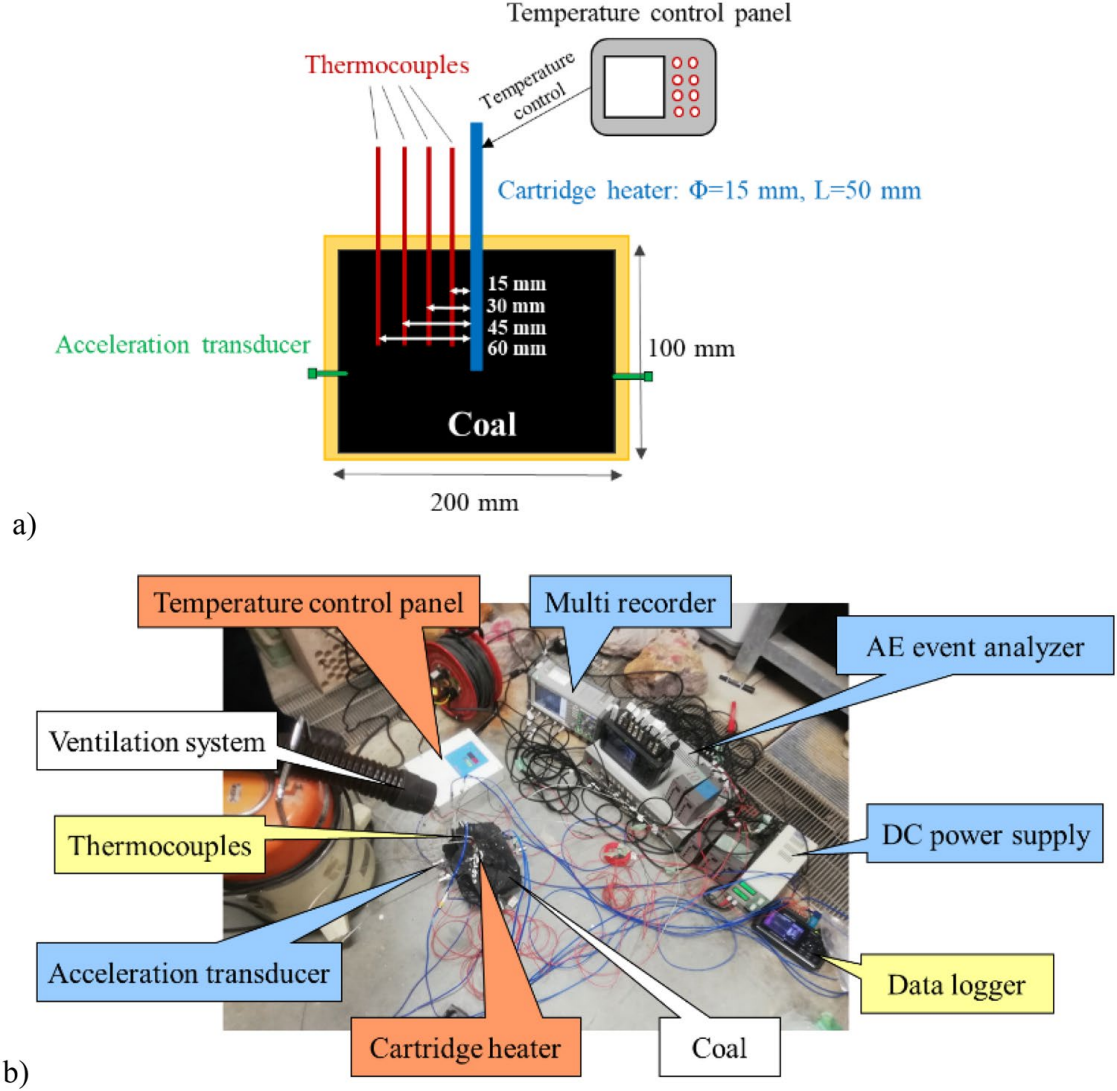
Experimental setup of the coal heating experiment: (**a**) Experimental design; (**b**) experimental system.

**Figure 2:**
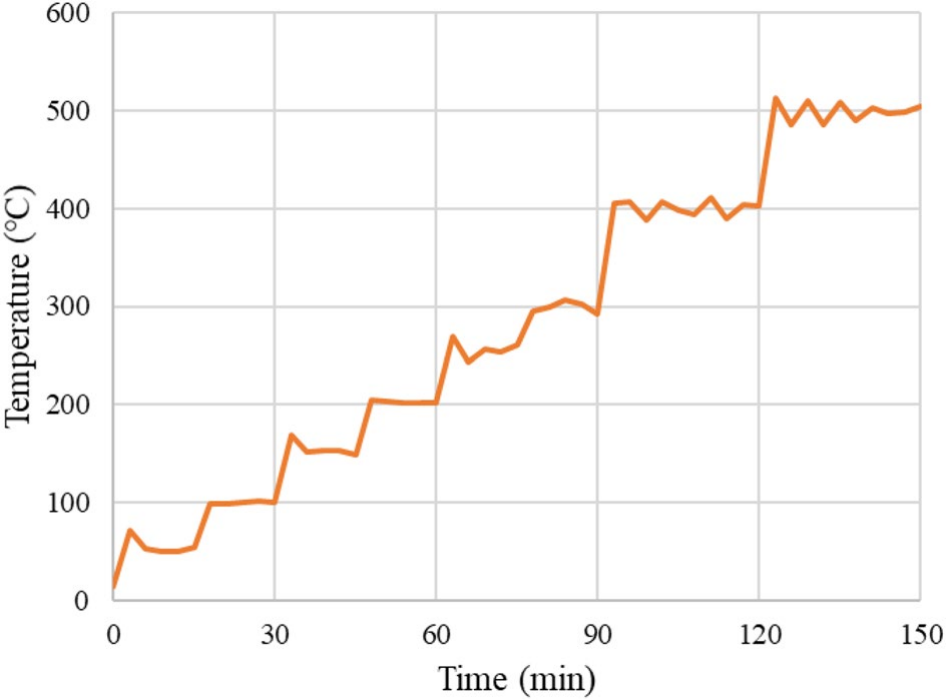
Temperature setup of a cartridge heater.

**Figure 3:**
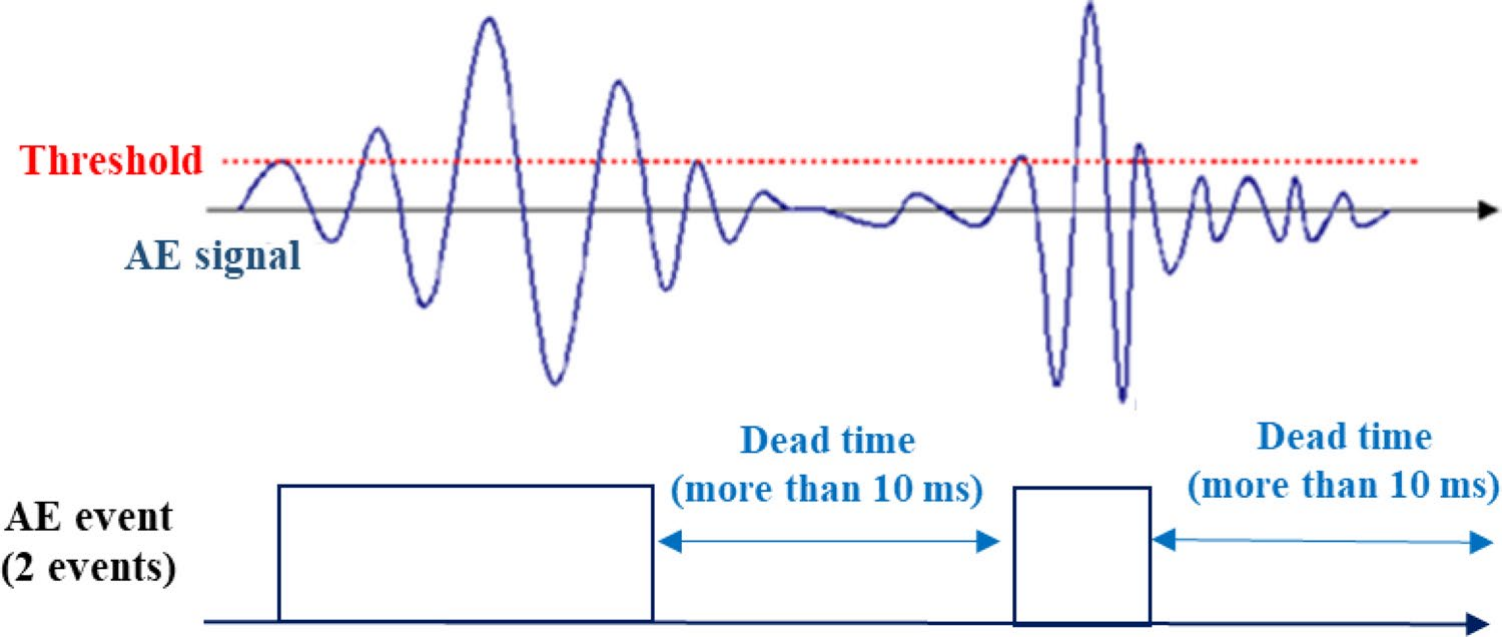
Definition of AE event with the dead time.

The coal heating experiment was carried out by using a coal block which is size of 200 mm × 200 mm × 100 mm. Table 1 shows the industrial and elemental analysis values of the coal. Coal is categorized as bituminous coal. Figure 1a,b show the experimental setup of the coal heating experiment. A cartridge heater whose diameter and length were respectively 15 mm and 50 mm was adopted to heat the coal. The temperature of the heater was controlled with a temperature control panel (YDC-15N; Yagami Corp.). The coal was heated by increasing the temperature of a cartridge heater every 50 °C up to 500 °C at 15 min intervals as shown in Fig. 2. Type K thermocouples (Chino Corp.) were installed at 15 mm intervals up to a distance of 60 mm from the cartridge heater. The 5 acceleration transducers (620HT; Teac Corp.) were installed on the end faces of the coal block to detect AE events. The acceleration transducers are charge type accelerometers with a frequency range of 1–10,000 Hz. The sensor has a diameter of 13.8 mm and a height of 29 mm. The number of AE events were counted in 2 acceleration transducers located at the different distance from a cartridge heater (90 mm and 110 mm away) by defining a threshold and a dead time as shown in Fig. 3. The threshold is 0.5 m/s^2^ and the dead time is set as 10 ms because the dead time is usually several ms in brittle materials: rock, concrete, and coal. The threshold is AE signal data was also recorded using a multi-recorder (GR-7000; Keyence Corp.) with a sampling time of 1 µs. The signals were filtered with a high-pass filter of 5 Hz and a low-pass filter of 10,000 Hz by the amplifiers (SA-611; Teac Corp.). AE signal data were used for AE source location analysis to discuss the relationship between the AE source and temperature distribution.

**Table 1:** Proximate and ultimate analyses of coal.

Calorific value (MJ/kg)	Proximate analysis (wt%)				Ultimate analysis (wt%)				
	Moisture	Ash	Volatiles	Fixed carbon	C	H	N	S	O
32.1	3.3	3.2	45.0	48.5	78.5	5.8	1.6	0.2	10.5

### Small-scale UCG experiment

**Figure 4:**
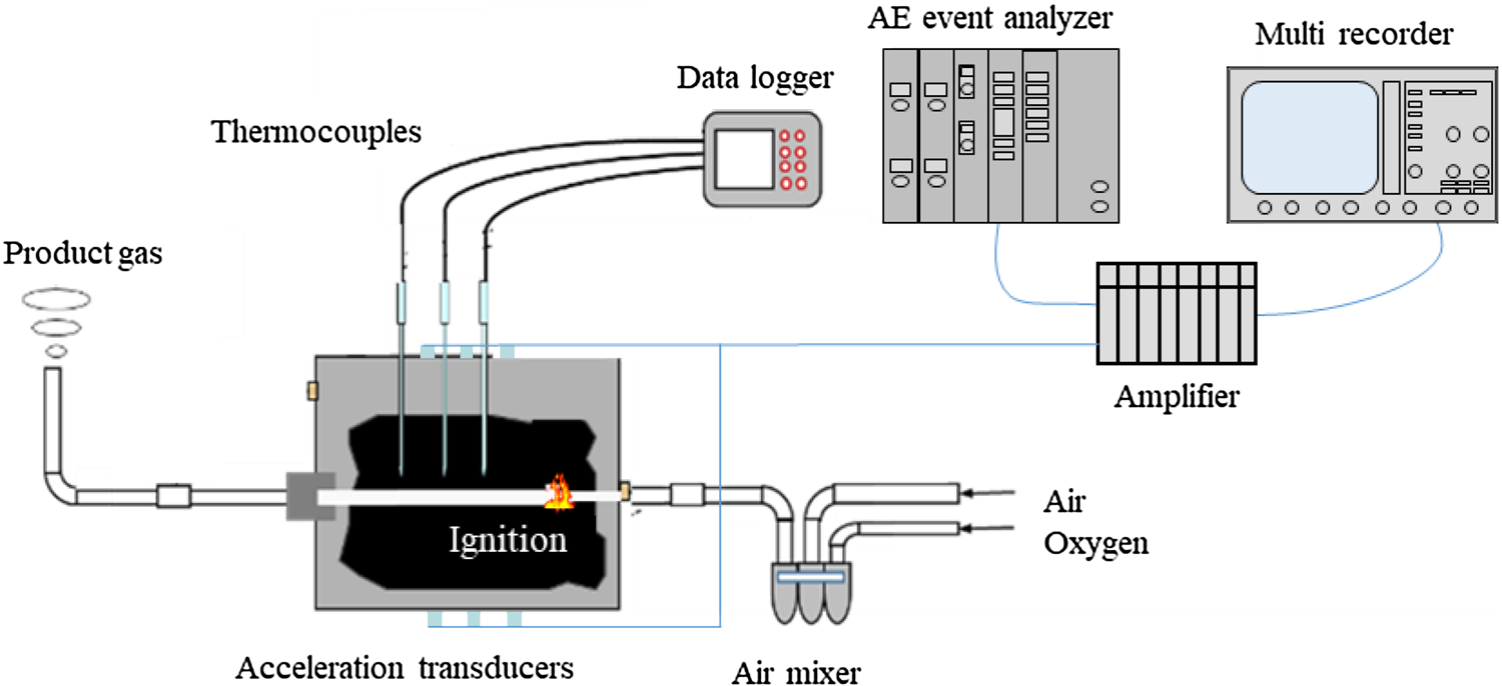
Small-scale UCG experiment.

**Figure 5:**
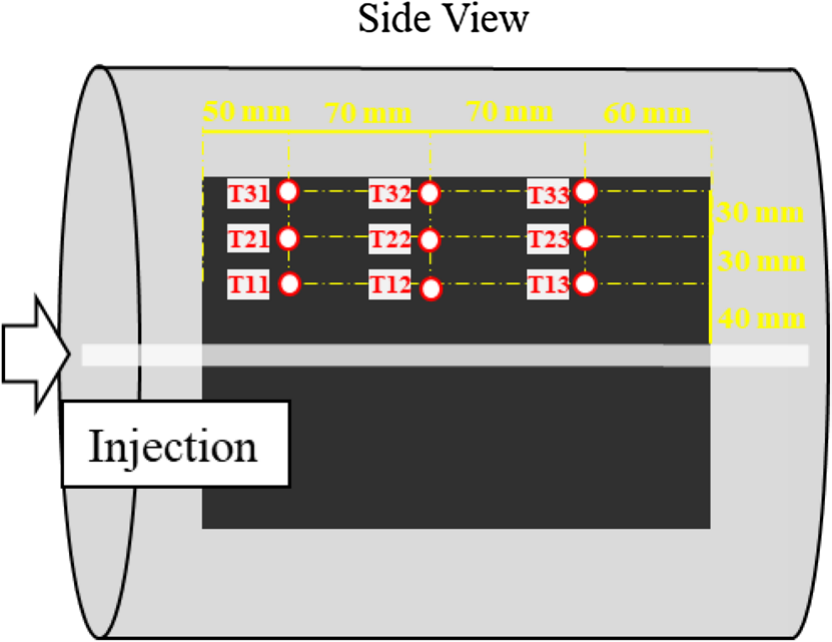
Distribution of thermocouples.

**Figure 6:**
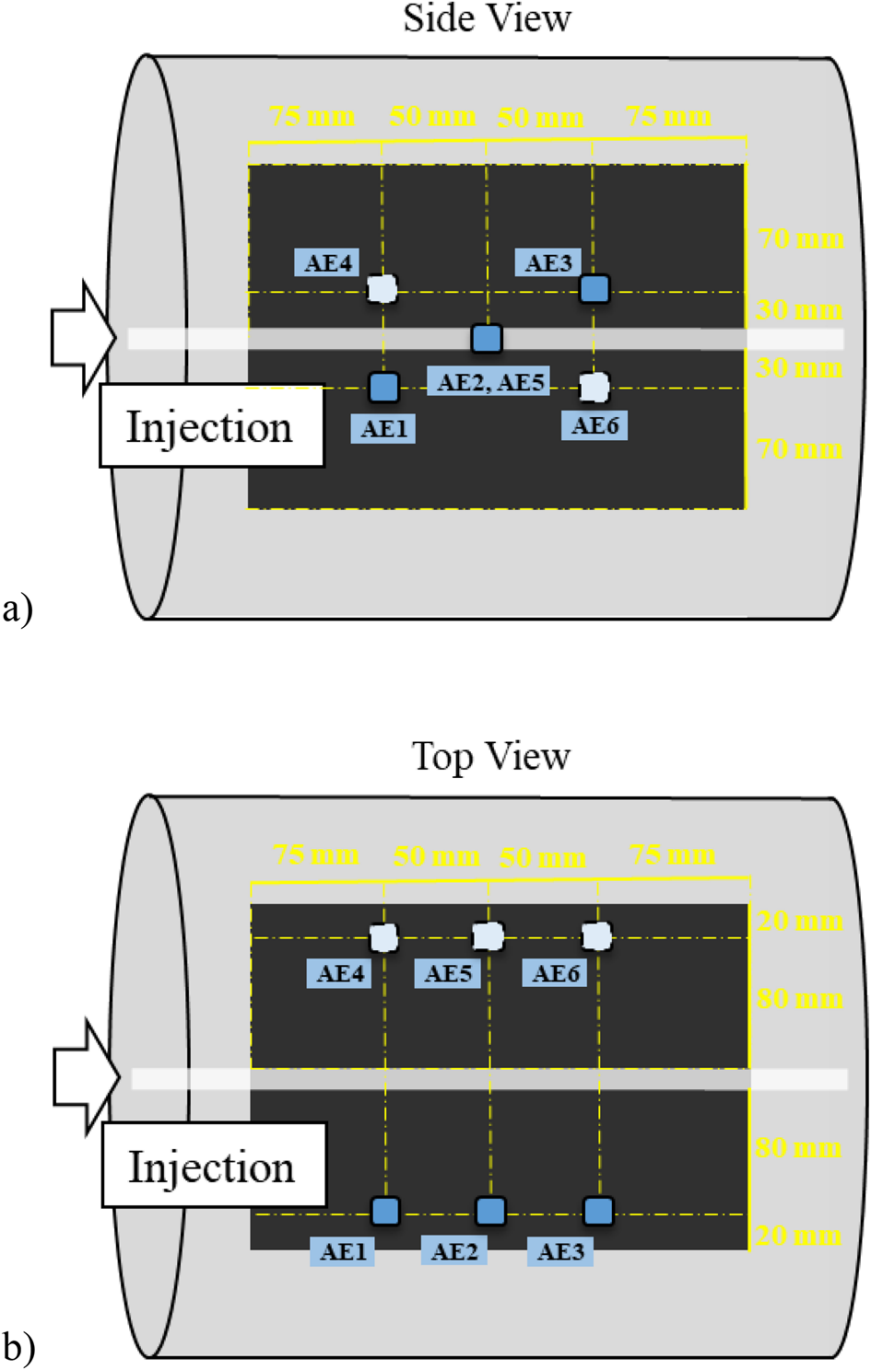
Distribution of acceleration transducers: (**a**) Side view; (**b**) top view.

The schematic diagram of the small-scale UCG experiment is shown in Fig. 4. In this experiment, the coal blocks whose size was 200 mm × 200 mm × 250 mm were prepared. The coal is same as in the coal heating experiment. A coal block was put in a metallic container whose size is 286 mm in diameter and 361 mm in height. Heat-resistant concrete was injected around the coal block to reduce gas leakage. A horizontal hole with a diameter of 20 mm was drilled in the center of the coal block as an ignition, injection, and production hole. During the UCG experiment, the temperature distribution of inside coal and the fractured events were monitored with the 9 thermocouples and 6 acceleration transducers, respectively. The acceleration transducers were attached to the coal brock by stainless steel waveguides of 6 mm in diameter and 100 mm in length; the holes for the waveguides were drilled with 6.5 mm in diameter from the surface of a metallic container to the coal block. The waveguides were fixed by a mixture of gypsum and cement. Figures 5 and 6 show the arrangement of each sensor. The gas burner was adopted in the ignition stage. After the ignition of coal is succeed, an oxidant mixture of air and oxygen was injected in order to maintain the gasification of the coal sample. The injection flow rate of the injectant was 10 L/min at constant and the oxygen concentration was 30%, 50%, and 70%. The duration of gasification was planned to be 6.7 h for 30% of oxygen concentration, 4.0 h for 50% of oxygen concentration, and 2.9 h for 70% of oxygen concentration in order to fix the total oxygen injection volume at 1200 L. However, it was 3.3 h when the oxygen concentration was 30% because the gasification did not continue. The product gas was analyzed every 15 min with the gas chromatograph (Micro GC 3000A; Inficon Co. Ltd.).

## Results and discussion

### Coal heating experiment

**Figure 7:**
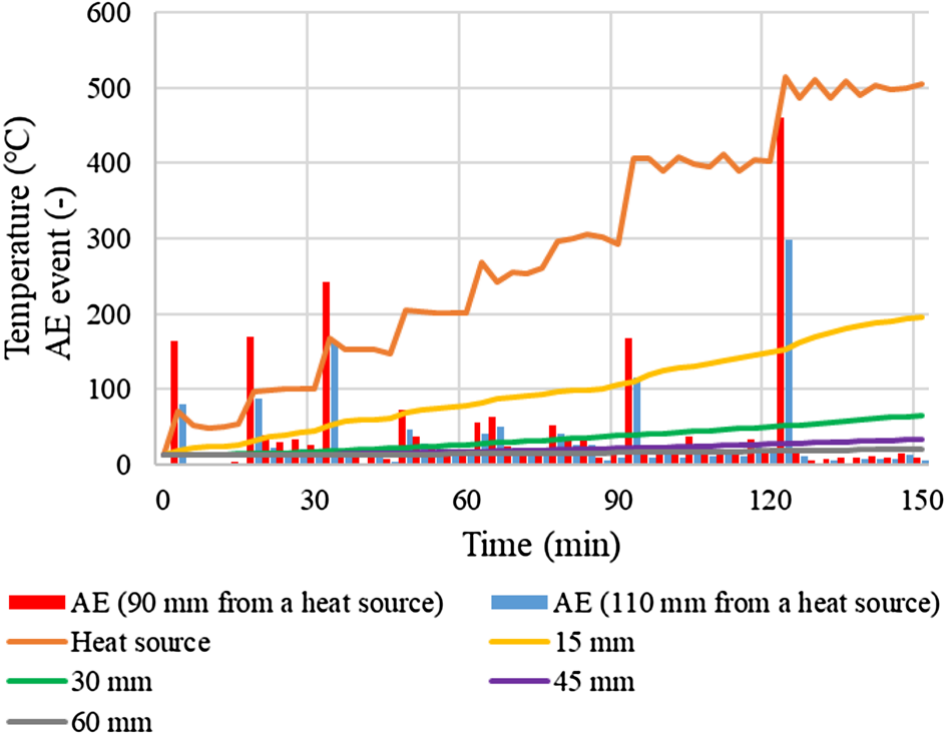
Temperature and AE events.

The results of temperature and AE events are respectively shown in Fig. 7. From the figures, it can be seen that the closer to the heat source, the higher the temperature of the thermocouple: the thermocouples of 15 mm, 30 mm, 45 mm, and 60 mm show respectively 197 °C, 68 °C, 38 °C, and 24 °C after the 150 min elapsed. The results of AE events show that many fracture events are generated when the temperature of the heat source changes dramatically. This indicates that the occurrence of AE activities is related to the temperature change of the coal, indicating that it is possible to understand the temperature change of the coal by AE monitoring. AE occurs around 50–150 °C and 400–500 °C. These AEs are generated due to the expansion of the gas-phase volume in the coal matrix, meaning that the fracturing activities should occur when the microstructures of coal are ruptured due to the increase of gas pressure. More AE events are detected around 400–500 °C. Coal gasification is generally promoted around 400 °C, meaning that fracturing activities around 400 °C may occur due to the gasification. Besides, Naka et al.^23^ described the deformation behavior of coal changes from expansion to contraction above 500 °C, indicating that the contraction behavior of the coal may affect the fracturing activity. In addition, more AE events can be detected the closer the distance from the heat source: the total AE events are respectively 2382 and 1515 in the sensor near the heat source (90 mm away from the heat source) and far from the heat source (110 mm away from the heat source). During coal heating, fracturing activities occur at several locations that exhibit high temperatures, meaning that AE events detected by the two sensors are hardly the same source. However, sensors closer to the heat source can detect AE events without being affected by attenuation because they are closer to the high-temperature area to generate fracturing events. This means that the AE events can be used to identify the location of the temperature change in the coal from the position of the sensors. However, the distribution of the location to generate the fracturing activities cannot be grasped from AE events. Therefore, AE source location analysis by multiple sensors is adopted in the next step.

**Figure 8:**
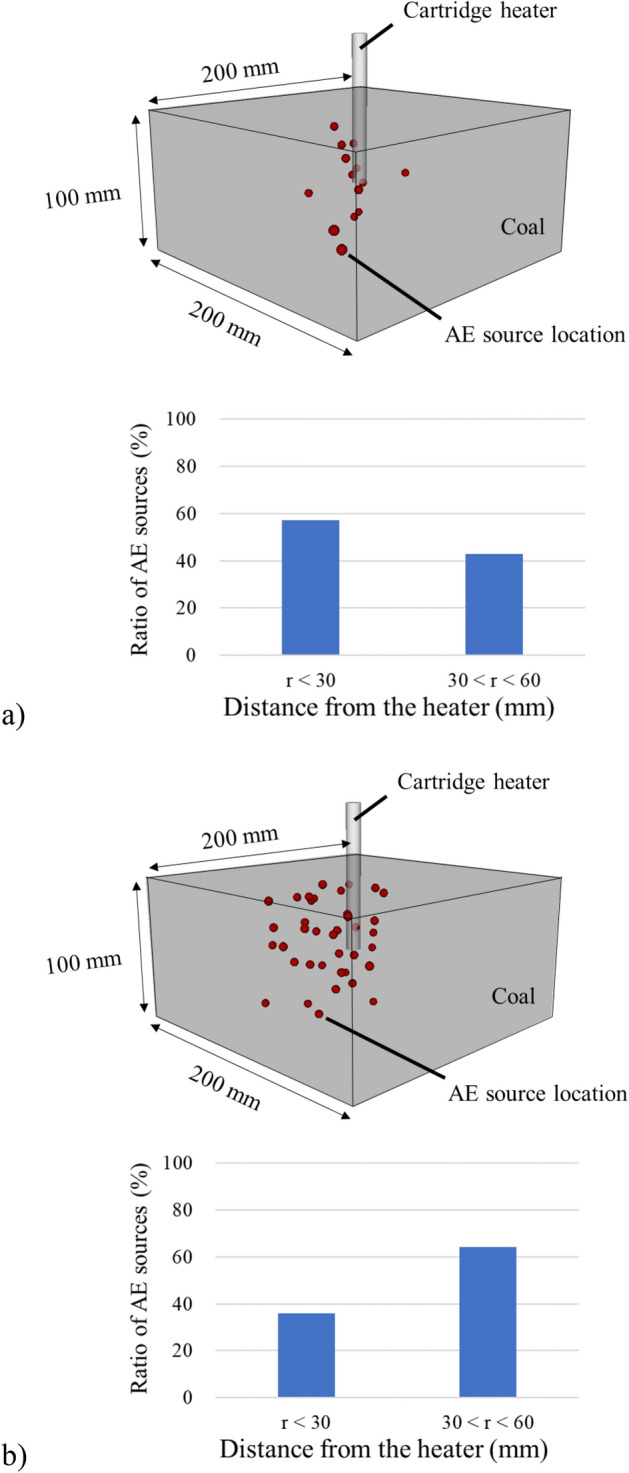
AE source location: (**a**) 0–45 min; (**b**) 90–150 min.

In this study, the source location of AE activities is calculated by the least-squares travel-time-difference method^32^. Figure 8a,b show the results of AE source location analysis and the ratio of source locations depending on the distance from the heat source when the temperature of the elapsed time is 0–45 min and 90–150 min. The dark gray color box shows the coal block and the cylinder in the center is a cartridge heater which is a heat source in the experiment. The red spheres depict the results of the AE source location. AE sources are dispersed from a heat source although the temperature dramatically changes near the cartridge heater. This result suggests that precise estimation of AE activities by coal heating is seemingly difficult due to the heterogeneity of coal, the properties changes of the coal with temperature changes, and other unknown factors. However, a rough estimation of AE activities can be done. The bar graph shows 57% of AE sources are located within the 30 mm distance and 43% of AE sources are located within the 30–60 mm distance from the heat source in 0–45 min. On the other hand, 36% of AE sources are located within the 30 mm distance and 64% of AE sources are located within the 30–60 mm distance from the heat source in 90–150 min. This means that the fracturing events are concentrated near the heat source in the initial stage while they are far away in the later stage of the experiment. This result is consistence with the temperature profile to expand the high-temperature area during the coal heating experiment, indicating that AE source location analysis enables monitoring the expansion of the high-temperature region.

### Small-scale UCG experiment

**Figure 9:**
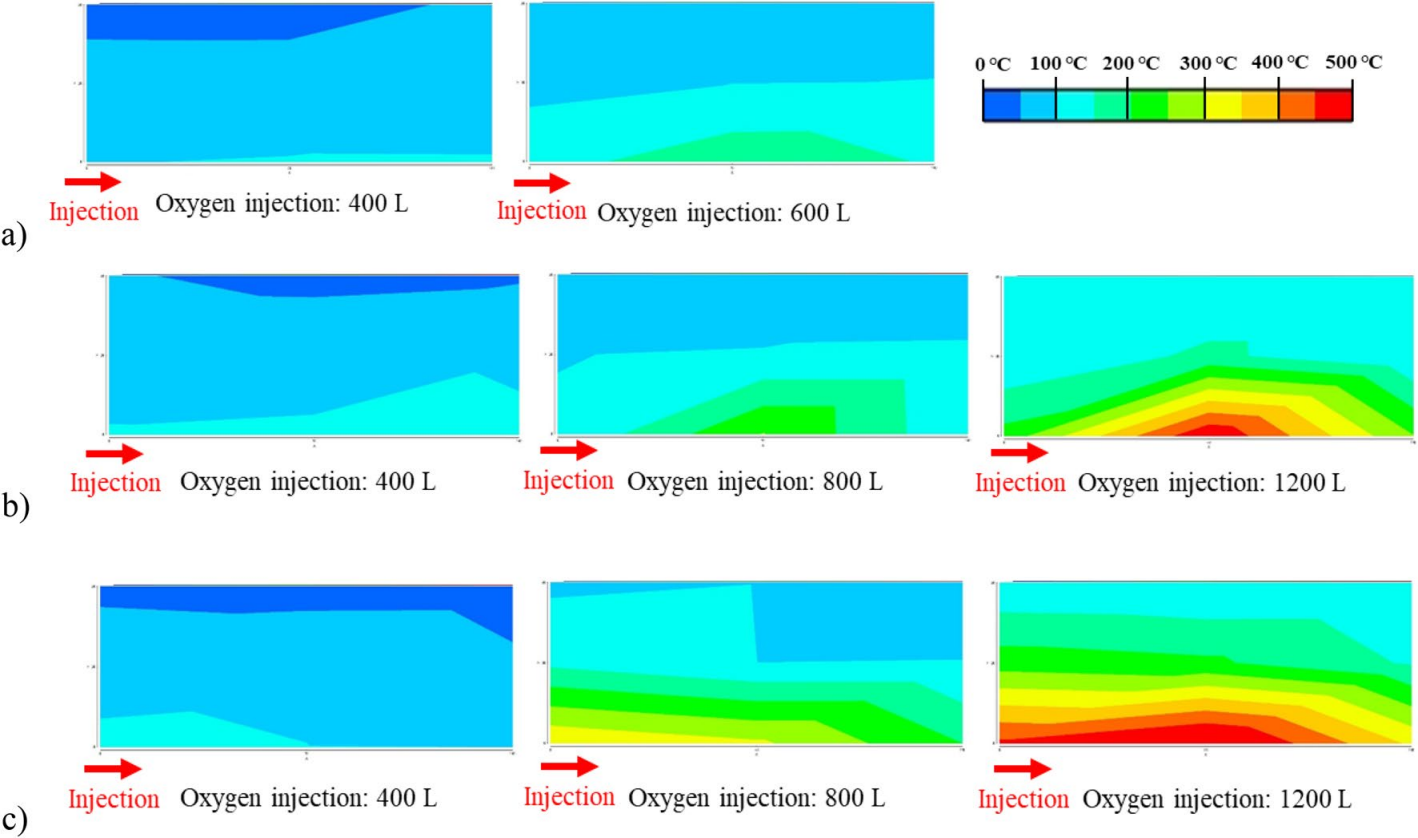
Temperature profile: (**a**) 30% oxygen concentration; (**b**) 50% oxygen concentration; (**c**) 70% oxygen concentration.

Figure 9a–c show the temperature profiles when the oxygen injection volume is 400 L, 800 L, and 1200 L, meaning that the elapsed time is 2.2 h (400 L) and 3.3 h (600 L) for 30% of oxygen concentration, 1.3 h (400 L), 2.7 h (800 L), and 4.0 h (1200 L) for 50% of oxygen concentration, and 1.0 h (400 L), 1.9 h (800 L), and 2.9 h (1200 L) for 70% of oxygen concentration. The figures are prepared based on the measurement data of thermocouples as shown in Fig. 5 and the contour range is divided in 50 °C intervals from 0 to 500 °C. They show the high-temperature region is formed on the injection side when the oxygen concentration is high. Although the cause of these phenomena is unknown, this may be because the reaction rate of the oxidation reaction is affected by the difference in oxygen concentration. In other words, the reaction rate of the oxidation reaction increased with increasing oxygen concentration, resulting in oxygen being consumed faster on the injection side under the condition of 70% oxygen concentration and a high-temperature region is formed on the injection side.

**Figure 10:**
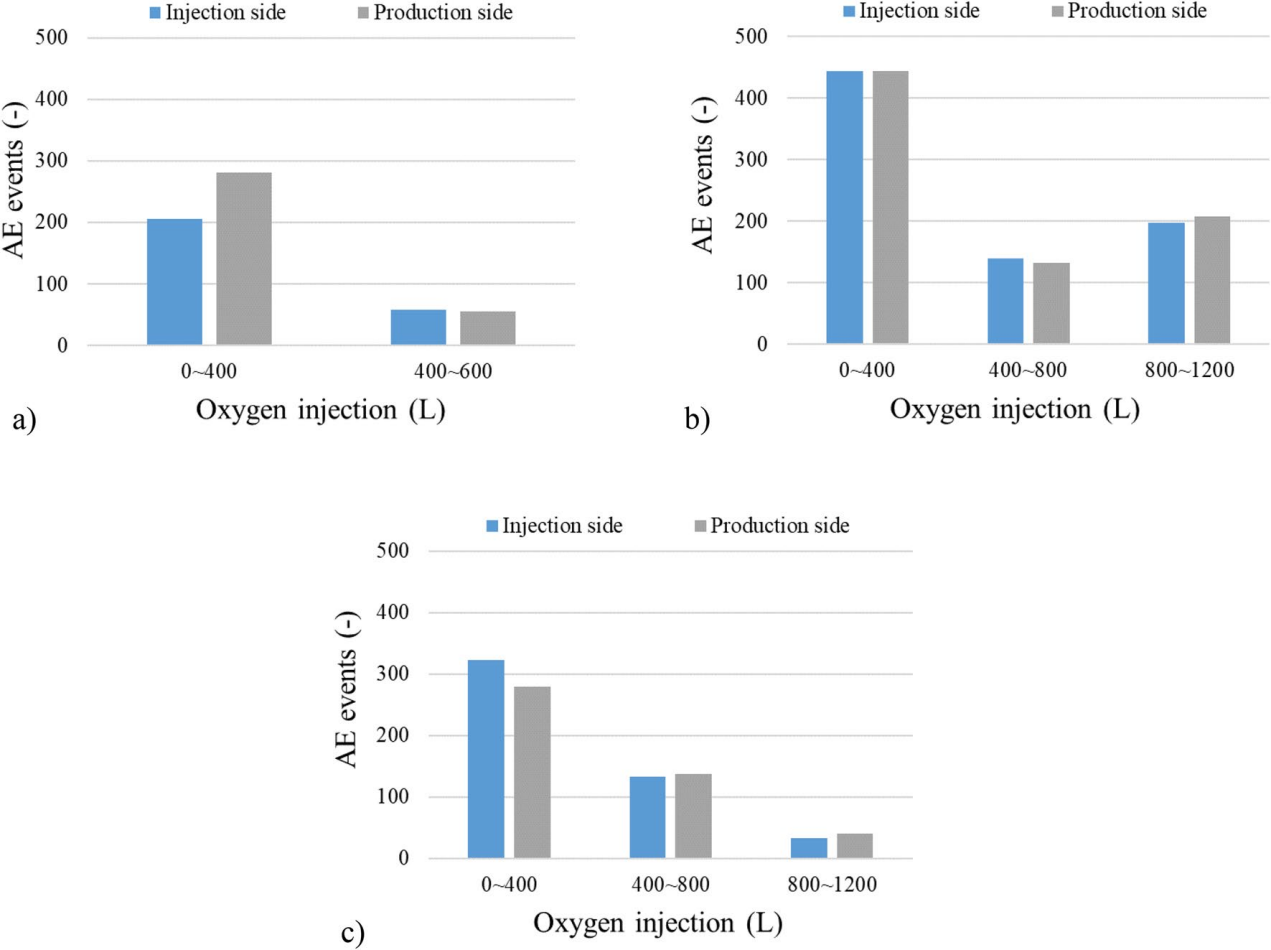
AE events: (**a**) 30% oxygen concentration; (**b**) 50% oxygen concentration; (**c**) 70% oxygen concentration.

**Figure 11:**
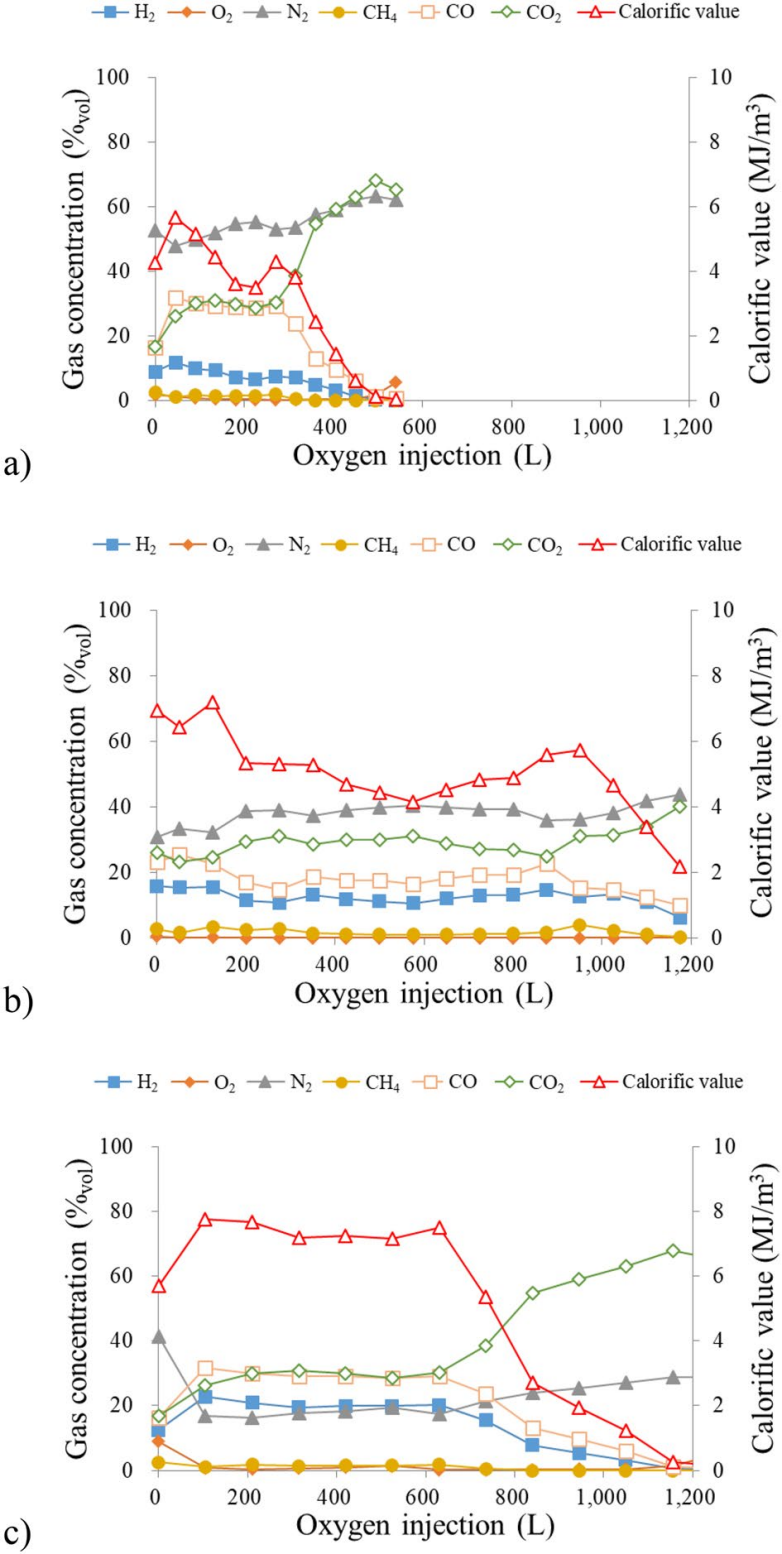
Product gas concentration: (**a**) 30% oxygen concentration; (**b**) 50% oxygen concentration; (**c**) 70% oxygen concentration.

Figure 10a–c show the results of the number of AE events for different oxygen concentrations. The AE events in AE1 and AE3 shown in Fig. 6 are used as the data of the injection/production side. AE events show the highest in the initial stage of the gasification regardless of the oxygen concentration. This means the fractured events are activated in the ignition process due to the drastic temperature changes and gas generation. The previous research has also confirmed the same tendency^33^. The AE events in the initial stage are higher on the production side for 30% of oxygen concentration while they are higher on the injection side for 70% of oxygen concentration. These results are in harmony with the temperature distribution results, indicating that many AE events occur at the locations where high-temperature regions are formed. The clear differences of AE events in the injection/production side are not found in the later stage of the gasification. This may be due to the small scale of the experiment, meaning that the distance between the acceleration transducers is too close to detect the difference of fracturing events for the wide gasification area. Wave attenuation occurs in proportion to the propagation distance. AE events cannot be detected due to wave attenuation if the distance from the fracturing source to the sensors is far. In the initial stage of the gasification, differences in the detected number of AE events occur depending on the location of the sensor because the high-temperature is small. On the other hand, the number of events does not show the difference in the sensors because wave attenuation does not occur due to the close distance between the sensors and the source of fracturing activities with the expansion of the high-temperature area. AE events increase during 800–1200 L of oxygen injection in 50% of oxygen concentration while they decrease with elapsed time in 30% and 70% of oxygen concentration. This can be explained by the gas production by the gasification reaction. Figure 11a–c show the product gas composition including the calorific value during the gasification. The calorific value has a downward trend with elapsed time in any oxygen concentration. This is because the molten slag which is generated from the ash contents of coal is inhibited to expand the gasification area due to the limitation of the gas–solid contact. The calorific value decreases dramatically after 360 L of oxygen injection in 30% of oxygen concentration and 840 L of oxygen injection in 70% of oxygen concentration. These results are consistence with AE events, meaning that AE events dramatically decrease after 400 L of oxygen injection in 30% of oxygen concentration and 800 L of oxygen injection in 70% of oxygen concentration. On the other hand, it increases from 4.87 to 5.73 MJ/m^3^ during 800–950 L of oxygen injection in 50% of oxygen concentration due to the increase of combustible gas components: H_2_ increases from 13.08 to 14.63%, CO increases from 19.13 to 22.52%, and CH_4_ increases from 1.30 to 4.00%. Besides, AE events also increase when the oxygen injection volume is from 800 to 1200 L. This finding suggests that the AE event is related to coal gasification. Therefore, AE monitoring is an effective way to evaluate the coal gasification in underground.

**Figure 12:**
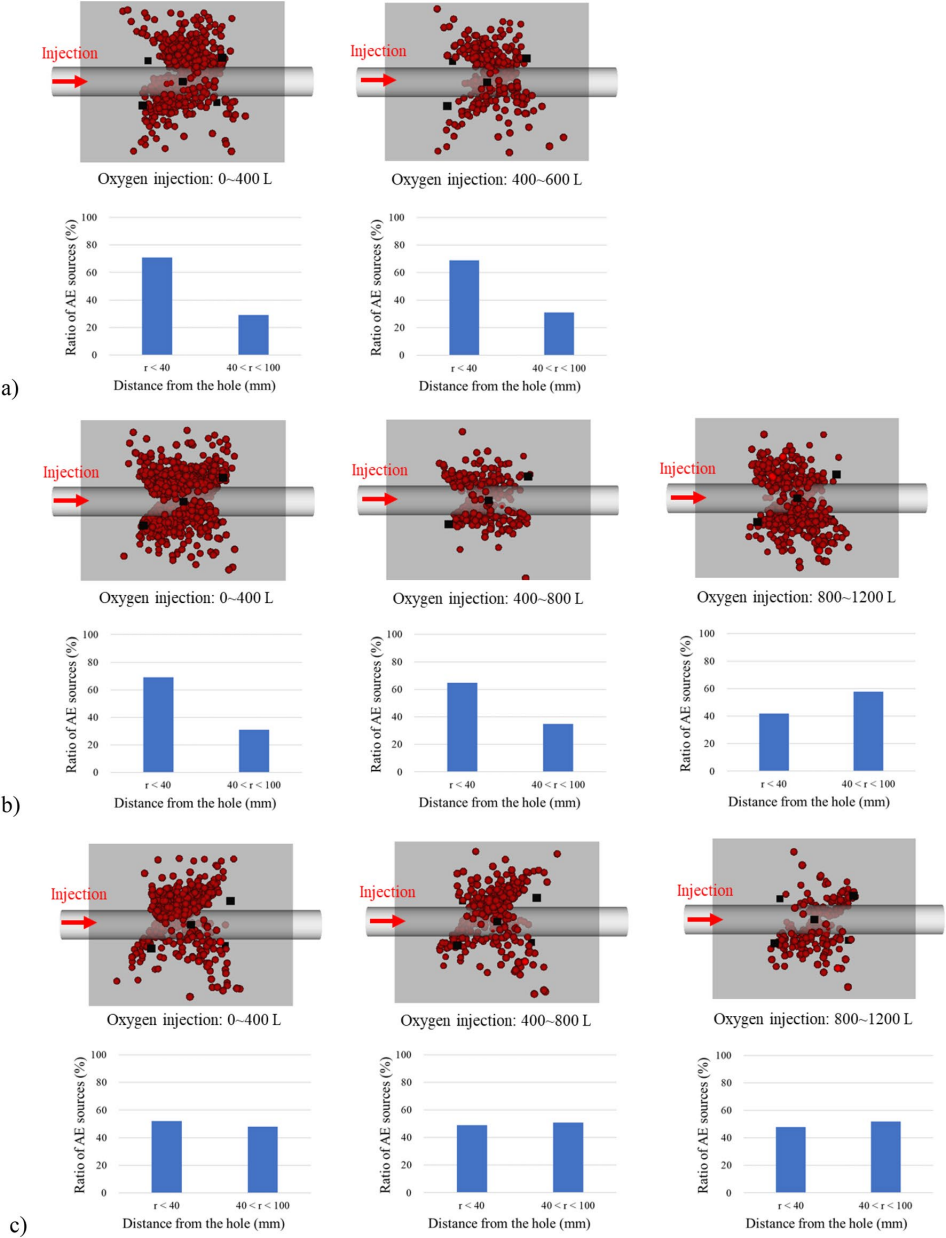
AE source location: (**a**) 30% oxygen concentration; (**b**) 50% oxygen concentration; (**c**) 70% oxygen concentration.

Figure 12a–c show the results of AE source location from the side view. The black square symbols indicate the acceleration transducers. AE sources are dispersed from a horizontal hole although the temperature dramatically changes near the hole, suggesting that the precise estimation of AE activities is difficult. However, the ratio of AE sources located far from the horizontal hole increases with the elapsed time in 50% and 70% of oxygen concentration: the ratio of AE sources located more than 40 mm away from the hole increases from 30%, 35%, and 54% when the oxygen concentration is 50% and increases from 40%, 42%, and 48% when the oxygen concentration is 70%. This result is consistent with the temperature profile to expand the gasification area, indicating that it is also confirmed AE source location analysis enables monitoring the expansion of the high-temperature region during the UCG process. The ratio of AE source location is the same regardless of the elapsed time in 30% of oxygen concentration: it is 69% within 40 mm from the hole and 31% in the area where the distance from the hole is more than 40 mm. This is because the gasification area does not expand widely in 30% of oxygen concentration. In actual UCG operations, it is difficult to directly measure the temperature in the coal seam, which can reach over 1000 °C. AE monitoring could be an effective technique for the estimation of the gasification area instead of temperature monitoring. This technique is also expected to be useful for controlling the gasification area by detecting the location of the gasification and its expansion when the injection condition changes.

## Conclusions

This study investigates the applicability of the AE technique as a monitoring system of the gasification area of UCG through the coal heating experiment and the small-scale UCG experiment. We have obtained the following knowledge.

- Many fracture events are generated when the temperature of the heat source changes dramatically, indicating that AE events are related to the temperature change of the coal.
- The closer the distance between the sensor and the heat source, the larger the number of AE events. This fact indicates that the location of the temperature change can be identified based on the position of the sensors.
- The high-temperature region differs based on the oxygen concentration. This may be because the reaction rate of the oxidation reaction; the oxygen is consumed faster on the injection side when the oxygen concentration is high.
- AE events increase especially during coal gasification.
- The expansion of the gasification area can be estimated by analyzing the AE source location.

## Data Availability

The data used to support the findings of this study are available from the corresponding author upon request.
